# Total intracorporeal laparoscopic ileal ureter replacement in a single position for ureteral stricture based on membrane anatomy

**DOI:** 10.1186/s12893-024-02363-w

**Published:** 2024-03-13

**Authors:** Guohao Wu, Haomin Li, Feng Luo, Handa Zheng, Yuanzhi Yuegao, Lishan Xie, Huilan Luo, Zhihui Chen, Dongming Ye, Caiyong Lai

**Affiliations:** 1 Department of Urology, The Sixth Affiliated Hospital of Jinan University, No. 88, Changdong Road, Dongguan, 523560 China; 2 Department of Urology, The First Affiliated Hospital of Jinan University, No. 613, Huangpu Road, Guangzhou, 510630 China; 3 Institute of Kidney Surgery, The First Affiliated Hospital of Jinan University, No. 613, Huangpu Road, Guangzhou, China

**Keywords:** Ileal ureteral replacement, Radiation-induced extensive ureteral strictures, Total intracorporeal laparoscopy, Reconstructive procedure

## Abstract

**Purpose:**

The aim of this study was to present our initial experience and prove the feasibility of total intracorporeal laparoscopic ileal ureter replacement (TILIUR) in a single position for ureteral stricture based on membrane anatomy.

**Materials and methods:**

Between January 2021 and April 2023, six patients underwent TILIUR in a single position for ureteral strictures based on membrane anatomy. All patients with a past medical history underwent radical hysterectomy with bilateral pelvic lymph node dissection as well as extensive ureteral stricture due to radiotherapy. The procedure is performed completely laparoscopically. Dissection of the digestive system as well as ureteral stricture or renal pelvis is based on membrane anatomy. The surgery is performed in a single position.

**Results:**

TILIUR in a single position for ureteral stricture based on membrane anatomy was successfully performed without open conversion in all patients. Among the 6 patients, 3 patients underwent combined ileal ureter replacement (IUR) and abdominal wall ostomy, 2 underwent unilateral IUR, and 1 underwent bilateral IUR. The mean length of the ileal substitution was 22.83 cm (range: 15–28). The average operative time was 458 ± 72.77 min (range 385–575 min), and the average intraoperative blood loss was 158 mL (range 50–400 mL). The median postoperative hospital stay was 15.1 d (range: 8–32). The median duration of postoperative follow-up was 15 months (range: 3–29 months). The success rate was 100%.

**Conclusions:**

TILIUR in a single position may be a promising option for ureteral stricture based on membrane anatomy in selected patients. Moreover, it has a positive effect on patients with renal insufficiency and urinary incontinence. Although IUR is difficult and risky, proficient surgeons can perform the procedure safely and effectively.

## Introduction

Cervical cancer (CC) is the fourth most common cancer among women worldwide, and radical treatment techniques include radical surgery and/or radiation. There is a high prevalence of adjuvant radiation therapy in CC [1, 2]; however, adjuvant radiation therapy significantly increases the risk of serious complications due to damage to the targeted tissues (pathological and normal) [3]. The treatment of radiation therapy-induced multisegmental strictures and even bilateral extensive ureteral strictures is more difficult and challenging due to the severe fibrosis and distortion of the anatomical planes caused by radiotherapy [4–6].

We previously reported that the membrane dissection concept of surgery has the advantage of a clear operative field with the protection of the ureteral blood supply by sheathing the free ureter [7]; here, we further applied the membrane dissection concept to the more complex situation of radiation-induced extensive ureteral stricture (RIEUS). The ileum is suitable as an alternative to the ureter because of its relatively rich blood supply and peristaltic nature [8]. Ileal ureter replacement (IUR) was first reported by Shoemaker in 1906 and was later popularized by Goodwin et al. [9] in the late 1950s.

Currently, most ileal substitution procedures are performed with the assistance of robotic systems, and a few are performed completely intracorporeally. In addition, data on total intracorporeal laparoscopic ileal ureter replacement (TILIUR) are poor and mainly limited to case reports. The present study reports our TILIUR technique in a single position, performed in six consecutive patients, and compares it with prior experiences available in the literature.

## Methods

### Patients

**Fig. 1:**
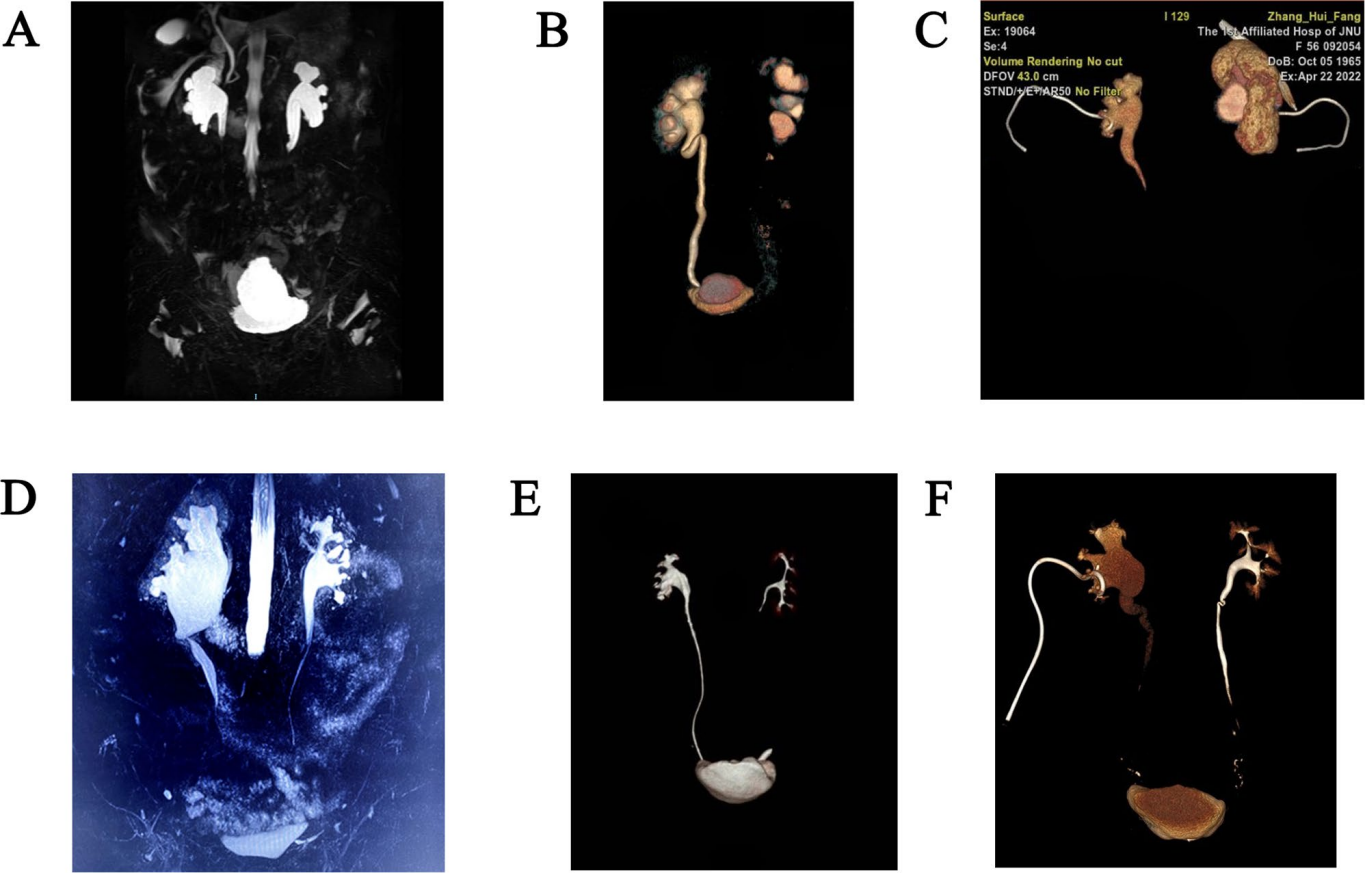
Imaging of the patient’s strictures prior to the surgery (**A**) MRU showed the mid-distal stricture of bilateral ureter, (**B**) Three-dimensional reconstruction of CTU showed long obliterating stricture of left tract and twisted upper right ureter with stricture, (**C**) Three-dimensional reconstruction of CTU showed long obliterating stricture of bilateral tract, (**D**) MRU showing left mid-ureteral stricture and right upper and mid-ureteral stricture, (**E**) Three-dimensional reconstruction of CTU showed mid-right ureteral stricture with indwelling ureteral stenting, (**F**) Three-dimensional reconstruction of the CTU showed the mid-distal stricture of right ureter

From January 2021 to April 2023, 6 patients treated with TILIUR in a single position were enrolled. The clinical data, including demographics, gynaecological cancer histories, perioperative records, imaging studies, complications, and follow-ups, were retrospectively collected. Subjective standards were used to make the diagnosis, such as flank pain, repeated fever and radiological criteria, including abdominal ultrasonography, retrograde urography or antegrade + retrograde urography, computed tomography urography (CTU) and magnetic resonance urography(MRU). All patients had ureteral stents placed and replaced regularly for strictures.According to our definition, the main surgical indication for TILIUR was patients with bilateral long or multiple ureteral strictures. TILIUR combined with abdominal wall ostomy is indicated in patients with bladder dysfunction, urinary incontinence, or preoperative creatinine greater than 2 mg/dL.Preoperative images of the patient with ureteral strictures are shown in Fig. 1.The Clavien‒Dindo classification system was used to evaluate the complications. Jinan University’s Affiliated Hospitals’ Ethics Committee (first and sixth hospitals) authorised this research. Informed consent was obtained from all participants.

**Fig. 2:**
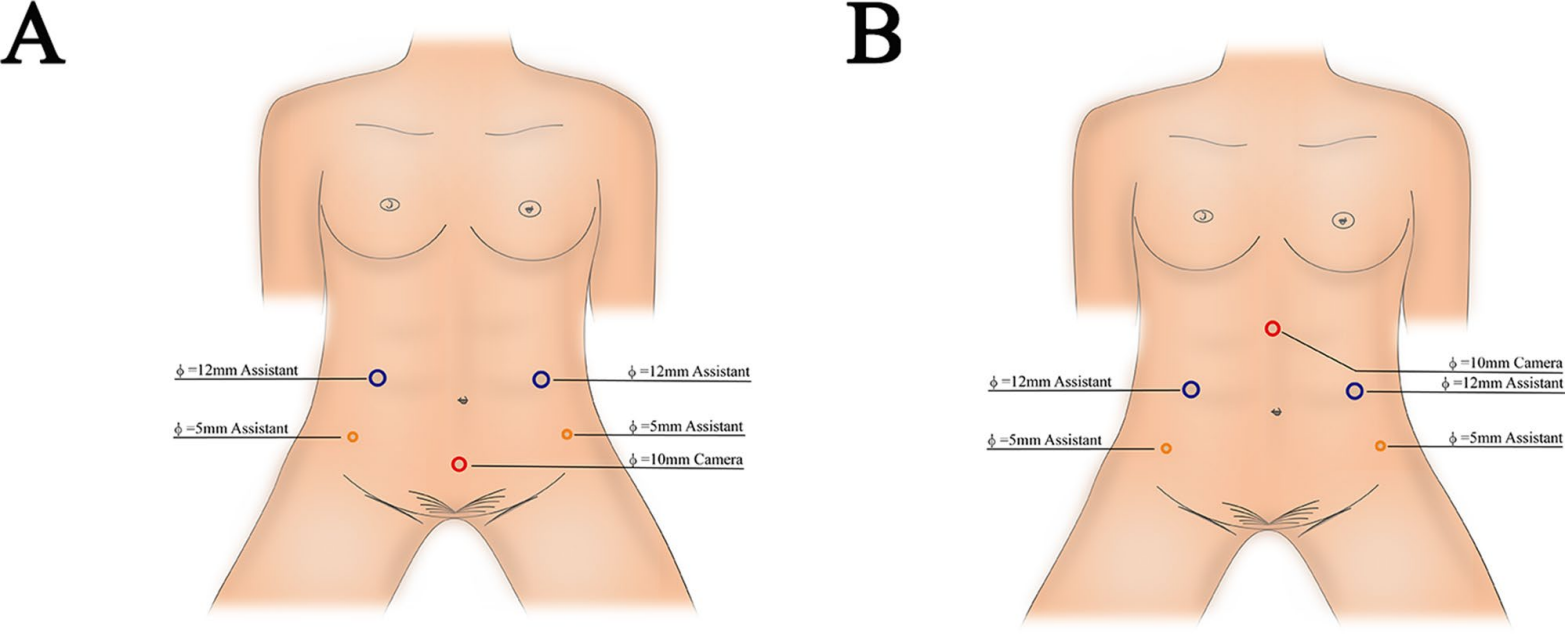
Position and trocar distribution (**A**) A five-port caudal approach, (**B**) A five-port cephalad approach

### Surgical technique

#### Preoperative preparation

Patients had a nephrostomy on the afflicted side one month before surgery to drain urine and protect renal function.Preoperatively, patients were routinely treated with third-generation cephalosporins for anti-infection treatment, which was adjusted after urine culture results were obtained if necessary. Patients were placed on a liquid diet for 2 days prior to surgery, the intestines were cleaned the day before the surgery with polyethylene glycol electrolyte, and enteral nutrition emulsions were taken to guarantee a scum-free diet.

#### Position and trocar distribution

After induction of general anaesthesia, a 20# three-way catheter was inserted. Each patient was placed in a 30° Trendelenburg tilt with both lower limbs spread outwards at approximately 60°. A five-port approach was used, and port placement is shown in Fig. 2A and B. A port (10-mm trocar) was placed midway between the umbilicus and the symphysis pubis (Fig. 2A) and midway between the umbilicus and the subxiphoid process (Fig. 2), and then a 30° camera was placed. The caudal approach was used for TILIUR combined with abdominal wall ostomy in a single position (Fig. 2A), and the cephalad approach was used in a single position for TILIUR (Fig. 2B).

### Surgical procedures

#### Dissection of the digestive system, ureteral stricture or renal pelvis based on membrane anatomy

##### Caudal approach

To expose the posterior peritoneum, the distal ileum was moved to the cephalic side. Then, the posterior peritoneum was incised along with the ileocecal and small bowel mesenteric roots at the fusion with the posterior abdominal wall. Dissection was performed by entering the level between the posterior mesenteric lobe and the anterior renal fascia, reaching the level of the right renal hilum. Subsequently, the renal fascia covering the surface of the right ureter was opened, the upper ureter was freed along the right ureteral sheath, and the distal ureter was clamped with Hem-o-lock clips at the ureteral stenosis. Then, the peritoneum was incised medially in the inferior mesenteric vein, and the level of the left renal hilum was revealed along with the level between the posterior lobe of the descending mesentery and the anterior lobe of the left renal fascia. The upper part of the left ureter was freed in the same way, and the distal stenotic ureter was clamped. Sterile saline (0.9%) was injected into the nephrostomy tube on the affected side to fill the urinary system to assess the proximal dilated ureter and dilated renal pelvis. By observing whether sterile saline flowed smoothly out of the proximal end of the ureter, the level of a healthy ureter was judged. Finally, the length of the ureteral defect was measured.

### Cephalad approach

Dissection of the digestive system and ureteral stricture via the cephalad approach was similar to that in our previously reported study [7]. On the left (or right) side, dissection was performed along the level between the posterior lobe of the left (or right) colonic mesentery and the anterior layer of the anterior renal fusion fascia, and the descending colon, the splenic flexure of the colon, the tail of the pancreas, or the right ascending colon was moved to the opposite side. Ureterolysis was carried out until proximal dilated normal ureteral tissue was encountered. If adjacent tissue adhesion was encountered, blunt and sharp dissection of dense adhesions was meticulously carried out, and the distal end of the dilated ureter was clamped with Hem−o−lock clips. The length of the ureteral defect was measured in the same way.

### Preparation of ileal ureter substitutes

**Fig. 3:**
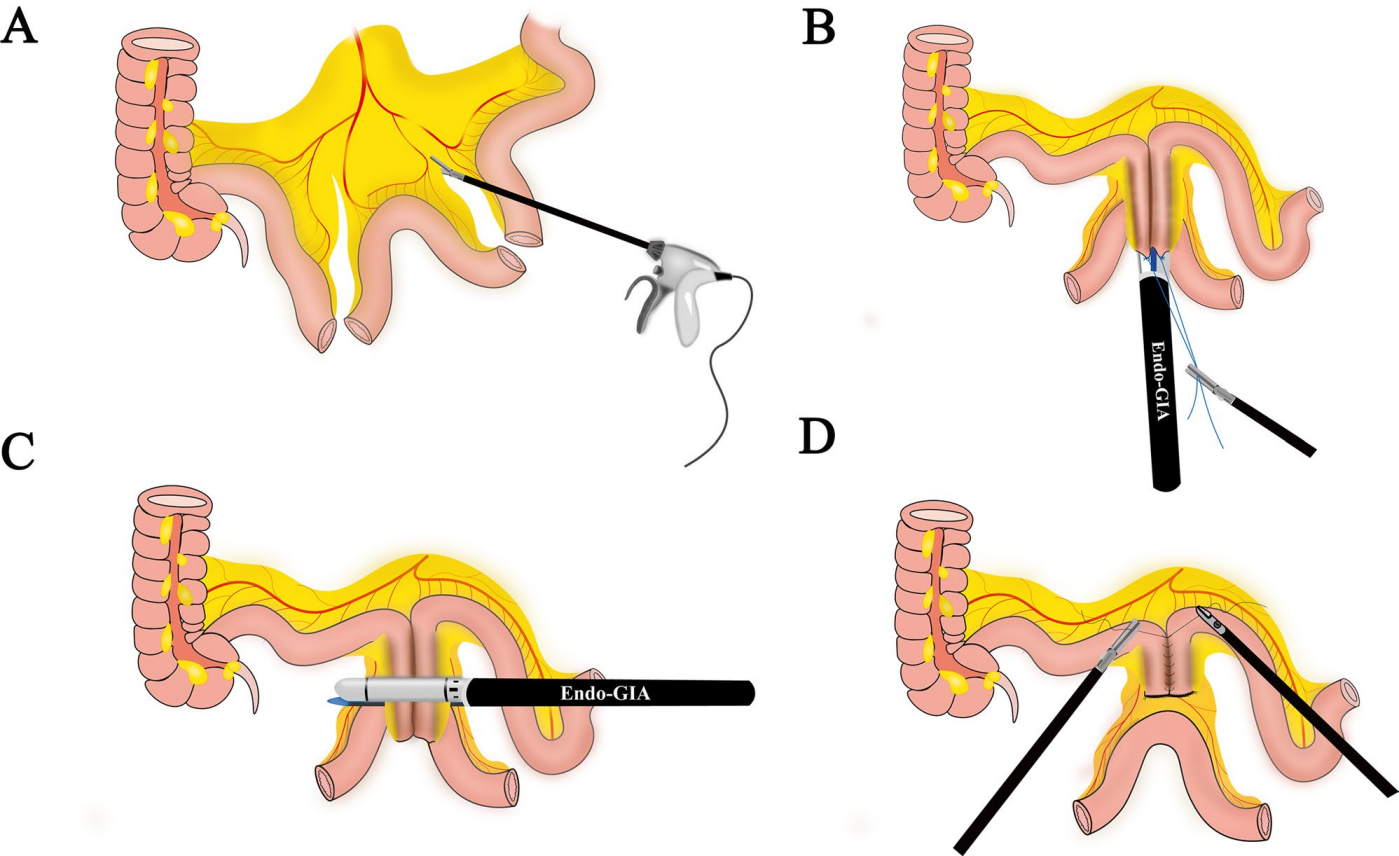
Preparation of ileal ureter substitutes (**A**) Measure and obtain the required length of the ileum, (**B**) and (**C**) Reconstruct the intestinal continuity, (**D**) Perform an interrupted anastomosis to strengthen the anastomotic edges

After measuring the length of the ureteral defect, a 20–30 cm ileal segment was marked and dissected using 3 − 0 absorbable thread at least 15 cm from the ileocecal region for backup (Fig. 3A). A longitudinal side-to-side anastomosis of the mesenteric rim was then performed using an Endo-GIA stapler (ECR60W, Ethicon Endo-Surgery, USA) to restore ileal continuity (Fig. 3B). Next, the proximal and distal ileum were anastomosed transversely with the assistance of an Endo-GIA stapler (Fig. 3C). Finally, the anastomotic edges were sutured interruptedly (Fig. 3D).

### Reconstruction of the ileal ureter replacement

The ileal ureteral replacement in this study was categorized into three different types.

**Fig. 4:**
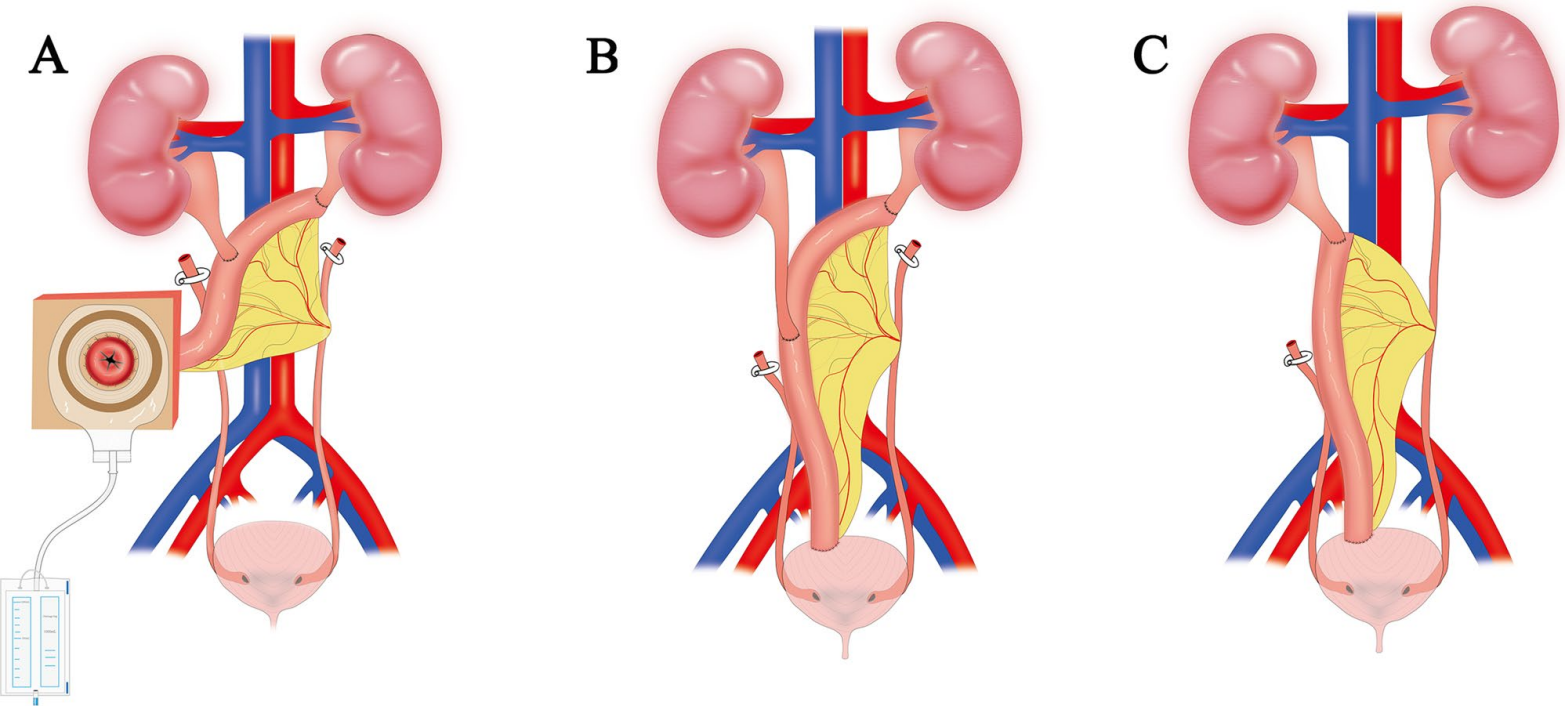
The types of IUR in this study (**A**) bilateral IUR combined with abdominal wall stoma, (**B**) reverse 7-shaped bilateral IUR, (**C**) unilateral IUR. IUR: ileal ureteral replacement

An F6 DJ tube was put in the left ureter after an end-to-end anastomosis between the proximal ileum and the left ureter using a 3 − 0 absorbable barbed suture.Another F6 DJ tube was placed into the ureter following side-to-side anastomosis between the right ureter and the middle ileum. Approximately 3 cm to the right of the umbilicus, a 2 cm diameter abdominal wall ostomy was made. The distal ileum was pulled out from the body and then twisted and sutured into a papilla shape before being implanted in the abdominal wall. Finally, an F22 latex catheter was left in the ileum(Fig. 4A).

In the bilateral IUR, called the reverse 7-shaped IUR, the proximal ileum was anastomosed end to end with the left ureter, the right ureter was anastomosed side to side with the ileum, and the distal ileum was twisted and sutured into a papilla shape and then implanted into the bladder (Fig. 4B).

For unilateral IUR, the proximal end of the ileum was end-to-end anastomosed with the right ureter, and the distal end of the ileum was twisted and sutured into a papilla shape and finally implanted into the bladder (Fig. 4C).

The tension-free, watertight, and full-thickness surgical principles were applied to all end-to-end anastomoses without torsion of the mesentery. A drainage tube was placed near the uretero-ileal anastomosis after confirming that the ureter was unobstructed and tightly anastomosed. The surgical incision was then stitched up.

### Follow‑up

Patients were followed up at 1, 3 and 6 months after surgery, and follow-up consisted of symptom evaluation, physical examinations, blood tests (including serum creatinine and electrolyte tests), routine urine tests, urological ultrasound, and CTU. The drain was removed when drainage was reduced to < 10 mL/day. Two weeks after the surgery, the three-way catheters were removed. DJ tubes were removed through cystoscopy two months after surgery. Surgical success was defined as the alleviation of subjective symptoms and the improvement of hydronephrosis.

## Results

A total of 6 female patients underwent TILIUR by the same surgeon between January 2021 and April 2023. All patients with medical history had radical hysterectomy and bilateral pelvic lymph node dissection. Table 1 shows patient demographics. The mean age was 56.7 y (range: 41–69). The main indication for ureteral replacement in our series was extensive ureteral strictures caused by radiotherapy after surgery for cervical cancer, with 4 cases of bilateral extensive ureteral strictures and 2 cases of right extensive ureteral strictures. Among the six patients, one patient each had flank pain as well as urinary incontinence with fever or haematuria, two had fever, and one patient each had flank pain and no symptoms. The mean time interval from radiotherapy to ureteral stricture detection was 23.7 months (range: 2–60).

**Table 1:** Demographics of the patients

No.	Age (years)	Sex	BMI (kg/m^2^)	Laterality, left/right/bilateral	Aetiology	Presenting symptoms	Time interval from radiotherapy to detection of ureteral stricture (months)	Management of preoperative ureteral stricture	Indication for surgery	Preoperative creatinine serum level (mg/gL)
1	56	Female	20.24	Bilateral	Gynaecologic surgery and radiation for cervical cancer	Flank pain, fever and UI	18	Bilateral double-J stent placement and PCN	BEUS	2.82
2	41	Female	22.92	Bilateral	Gynaecologic surgery and radiation for cervical cancer	Flank pain, haematuria and UI	2	Bilateral double-J stent placement and PCN	BEUS	1.50
3	56	Female	28.60	Bilateral	Gynaecologic surgery and radiation for cervical cancer	Fever	24	Bilateral double-J stent placement and PCN	BEUS	1.13
4	69	Female	24.61	Bilateral	Gynaecologic surgery and radiation for cervical cancer	Asymptomatic	60	Bilateral double-J stent placement and PCN	BEUS	3.69
5	58	Female	30.20	Right	Gynaecologic surgery and radiation for cervical cancer	Flank pain	24	Right double-J stent placement and PCN	REUS	0.81
6	60	Female	18.83	Right	Gynaecologic surgery and radiation for cervical cancer	Fever	14	Right double-J stent placement and PCN	REUS	0.75

PCN, percutaneous nephrostomy; BEUS, bilateral extensive ureteral strictures; REUS, right extensive ureteral strictures; UI, urinary incontinence

**Fig. 5:**
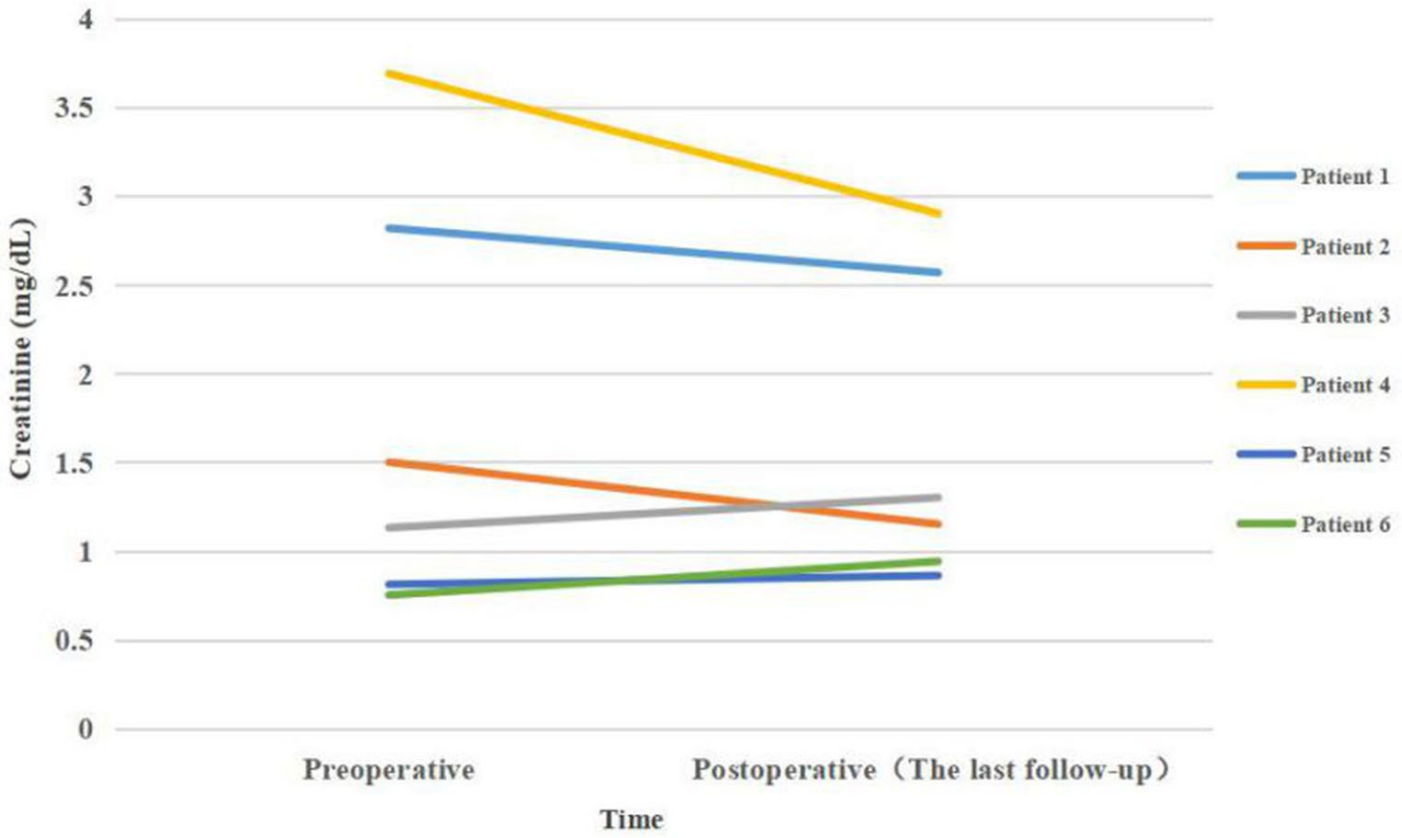
The trends of creatinine before and after surgery for each patient

All patients had a double-J stent and percutaneous nephrostomy for preoperative ureteral stricture.Table 2 shows patient surgery and follow-up data. The mean length of the ileal substitution was 22.83 cm (range: 15–28). The average operative time was 458 ± 72.77 min (range 385–575 min), and the average intraoperative blood loss was 158 mL (range 50–400 mL). Among the 6 patients, 3 patients received combined IUR and abdominal wall ostomy, 2 received unilateral IUR, and 1 received bilateral IUR. Each type of IUR is shown in Fig. 3. The median postoperative hospital stay was 15.1 d (range: 8–32). The median duration of postoperative follow-up was 15 months (range: 3–29 months). Regarding postoperative complications, one patient experienced incomplete ileus, which was resolved after conservative treatment, and one patient had retraction of the abdominal wall stoma due to abdominal obesity. In total, 6 patients had a decreased or stable hydronephrosis.The average creatinine levels before surgery and at the last follow-up visit were 1.78 (0.75–3.69) mg/dL and 1.62 (0.86–2.90) mg/dL, respectively. Renal function improved or remained stable in all 6 patients (Fig. 5).

**Table 2:** Surgical details and follow-up data

No.	Length of the ileal ureter(cm)	Operative time (minutes)	Estimated blood loss(mL)	Surgical technique	Surgical complication (Clavien classification)	Postoperative hospital stay(days)	Creatinine serum level at last follow-up(mg/dL)	The state of hydronephrosis	Follow-up (months)
1	20	575	100	Reverse 7-shaped + abdominal wall ostomy	None	9	2.57	Left side:mild;right side:mild^#^	29
2	28	425	100	Reverse 7-shaped + abdominal wall ostomy	None	8	1.15	Left side:mild;right side:mild^##^	26
3	27	390	50	Reverse 7-shaped	None	9	1.30	Left side:mild;right side:mild^##^	13
4	20	490	400	Reverse 7-shaped + abdominal wall ostomy	Grade I, abdominal wall stoma retraction	32	2.90	Left side:moderate;right side:moderate^#^	17
5	27	485	100	Right IUR	None	13	0.86	Right side:mild^##^	12
6	15	385	200	Right IUR	Incomplete ileus	13	0.94	Right side:none^##^	3

IUR, ileal ureter replacement;#: Patient with renal insufficiency, shown with ultrasound results; ##: Patient with normal renal function and shown with CTU results

## Discussion

The ischaemia and fibrosis of the ureter and surrounding tissues caused by radiotherapy is a great challenge for surgeons. Long ureteral defects are treated surgically by constructing a nonrefluxing and nonobstructed urine outflow as quickly as feasible to restore or stabilize renal function [10].

In our case series, all patients had ureteral stents placed and replaced regularly for strictures. However, long-term indwelling ureteral stents may cause recurring urinary tract infections and renal function issues, putting patients under severe emotional, psychological, and financial stress.It is well known that the treatment of extensive long-segment ureteral strictures includes uretero-ureterostomy, renal autotransplantation, Boari bladder flap, and IUR. Historically, IUR was performed for ureteral strictures secondary to tuberculosis. However, among all available treatment options for radiation-induced extensive ureteral strictures, IUR is the last and only viable option at this time. Park JJ et al. [11]and Monn MF et al. [5] reported their experience with IUR in the treatment of radiation-induced ureteral stricture, but their procedures were performed open. Limited literature has reported completely intracorporeal laparoscopic IUR, and only a few case reports have concentrated on the treatment of long ureteral strictures after radiation therapy.

For example, Kochkin A et al. [12] reported the experience of 40 cases of total intracorporeal laparoscopic IUR for the treatment of long ureteral defects, which required a change in table position during the procedure. In addition, it has been reported that the current robot used to perform IUR must be undocked and redocked during the ileovesical anastomosis stage of the procedure [4]. Furthermore, ureteral strictures caused by radiation therapy after surgery for cervical cancer are often associated with severe scarring and fibrosis of the periureteral tissue, which poses a serious challenge for the surgeon when dissecting the ureter while preserving its blood supply. To optimize the problems faced above, we innovatively proposed a single-position TILIUR, and put the concept based on membrane anatomy that we reported earlier, that is, pay more attention to the plane between tissue and tissue when dissecting the ureter, and applied this technique to the dissection of ureteral strictures after radiotherapy.

The standard treatment for early-stage cervical cancer is radical hysterectomy with bilateral pelvic lymph node dissection. The important technical step in radical hysterectomy is to perform wide dissection of the periureteral tissue and bladder [13].Thus, we can consider that the ureters, bilateral iliac vessels, and peripheral tissues of the bladder in these patients after radical hysterectomy are mostly “naked” because of bilateral pelvic lymph node dissection, dissections of the ureters, and adjuvant radiation therapy and are replaced by a covering of fibrous scar tissue. It is difficult to free the fibrous scar tissue covering the surface of the ureter, and an alternative strategy was employed in our study series by looking for normal tissue with space as an entry point and by a combination of blunt and sharp methods if severe fibrous scar tissue was encountered.

Thereare limited TILIUR reports for treating postoperative cervical cancer radiotherapy-induced ureteral strictures. Gözen AS et al. [12] reported 40 cases of TILIUR in 2020, one of which was for ureteral strictures caused by cervical surgery followed by radiotherapy, and the mean operative time in this series was 335 (150–680) minutes, with a mean estimated blood loss of 221 (50–400) ml; Li B et al. [14]reported two cases in 2021, with operative times of 420 min and 410 min per patient and an estimated blood loss of 120 ml and 100 ml, respectively; Li X et al. [15] reported 15 cases of robotic-assisted total intracorporeal robot-assisted ileal ureter replacement (RA-IUR) in 2023, with 7 patients treated with unilateral RA-IUR and 8 patients treated with bilateral RA-IUR, with a mean operative time of 261.8 min (183–381 min) and an estimated blood loss of 64.7 ml (30–100 ml), including 7 patients who had surgery for cervical cancer plus radiotherapy. Our series were all in this category, with a mean operative time of 458 min (385–575 min) and an estimated blood loss of 158 ml (50–400 ml). Although the operation time was slightly longer and the estimated blood loss was slightly higher, in terms of postoperative outcome, the results were generally consistent with the above studies. One patient with postoperative incomplete ileus recovered following conservative therapy, one with abdominal wall stoma retraction due to obesity, and the others experienced no complications.

Next, in terms of indications for surgery, the main surgical indication for TILIUR is patients with bilateral long or multiple ureteral strictures, which is similar to the surgical indications reported in the current literature [15]. IUR combined with abdominal wall stoma is recommended for patients with bilateral long or multiple ureteral strictures accompanied by bladder dysfunction, such as urinary incontinence, or preoperative creatinine greater than 2 mg/dl. Because gynaecological cancer surgery and radiotherapy can cause complications of low-compliance bladder and vesicovaginal fistula, in this case, it can cause repeated urinary tract irritation or urinary incontinence. To maintain kidney function and improve lower urinary tract symptoms at the same time, urinary tract diversion is inevitable [16, 17].

Although the mid-urethral sling is the gold standard for treating urinary incontinence in women [18], for the loss of bladder capacity and urethral sphincter function that cannot be repaired, abdominal wall ostomy is an appropriate and feasible surgical method.

A previous study also supports the fact that half of the patients with serum creatinine over 2 mg/dl develop hyperchloremic metabolic acidosis, and the procedure needs to be changed to the conduit [19]. However, a study by Armatys SA et al. [20]showed that six patients had preoperative baseline creatinine greater than 2.0 mg/dl, and renal function stabilized or improved in five cases; therefore, we believe that IUR in patients with preoperative creatinine greater than 2.0 mg/dl still has a role in stabilizing renal function. Impaired renal function was not a contraindication to surgery, according to studies, and IUR may help preserve renal function in patients with high serum creatinine (> 2 mg/dl) [21, 22]. In our case, two patients with preoperative creatinine greater than 2.0 mg/dl had a decrease in creatinine at the last follow-up compared with the preoperative period.

Based on our encouraging initial experience, TILIUR in a single position based on membrane anatomy may be a promising option for the treatment of ureteral strictures induced by radiotherapy for cervical cancer. There are certain considerations in the management of RIEUS based on our experience. First, for patients with acute renal failure caused by RIEUS, preoperative percutaneous nephrostomy protects renal function and reduces the risk of urinary tract infection as well as effectively decreasing the incidence of postoperative urinary leakage and improving the healing of the ureter-ileum anastomosis; moreover, nephrostomy can be used intraoperatively to observe saline flow to assess the health of the ureter. Second, for patients with RIEUS accompanied by renal insufficiency and bladder dysfunction, TILIUR combined with abdominal wall ostomy may be considered because of the positive effect on the protection of renal function. All anastomoses adhered to the surgical principles of being tension free and waterproof and protecting the blood supply. Therefore, this requires proficiency in laparoscopic techniques as well as extensive experience in ureteral reconstruction for the urologist. Third, for dissection of the bilateral renal pelvis and upper ureter, we used a single caudal approach. This approach offers the advantages of avoiding procedures such as changing positions, resterilization, and draping during the surgery as well as simplifying the process and significantly reducing the operating time.

Although encouraging, the results of our report must be considered in the context of its limitations. First, all of the procedures were carried out by surgeons who were well trained and had much expertise with laparoscopic surgery. As a result, our findings could not be applied to all surgeons. Additionally, this was a pilot study, and the sample size was limited, which might impact the results. Therefore, to confirm our preliminary findings, more research with larger populations and longer follow-ups is needed.

## Conclusions

TILIUR in a single position for ureteral stricture based on membrane anatomy may be a promising option for selected patients. Moreover, it has a positive effect on patients with renal insufficiency and urinary incontinence. Although IUR is difficult and risky, proficient surgeons can perform the procedures safely and effectively.

## Data Availability

All data generated or analysed during this study are included in this article.

## Abbreviations

TILIUR: Total intracorporeal laparoscopic ileal ureter replacement
IUR: Ileal ureter replacement
CC: Cervical cancer
RIEUS: Radiation-induced extensive ureteral stricture
CTU: Computed tomography urography
